# Supplementary material for: Ligand discrimination and gating in cyclic nucleotide-gated ion channels from apo and partial agonist-bound cryo-EM structures
Source: eLife. 2018 Jul 20;7:e39775. doi: 10.7554/eLife.39775 (PMC6093708; doi:10.7554/eLife.39775)
Supplement: Supplementary file 1. [file elife-39775-supp1.docx]

|  | Apo-SthK | cAMP-SthK | cGMP-SthK |
| --- | --- | --- | --- |
| **Data collection/processing** |  |  |  |
| Microscope | Krios/GIF/K2 | Krios/K2 | Krios/GIF/K2 |
| Voltage (kV) | 300 | 300 | 300 |
| Magnification | 105000 | 22500 | 105000 |
| Defocus (µm) | -1.0 – -2.2 | -1.0 – -2.2 | -1.0 – -2.2 |
| Pixel size (Å) | 1.0961 | 1.07325 | 1.0961 |
| Total dose (e^-^/Å^2^) | 52 | 70 | 52 |
| Exposure time (s) | 8 | 10 | 8 |
| Number of frames per micrograph | 40 | 50 | 40 |
| Dose per frame (e^-^/Å^2^) | 1.3 | 1.4 | 1.3 |
| Number of micrographs | 2130 | 5473 (823, 2210, 2440) | 2744 (1404, 1340) |
| Number of particles after 2D classification | 179504 | 572844 | 199965 |
| Final particles | 51115 | 81501 | 91800 |
| Resolution C4 (unmasked, Å) | 4.07 | 3.71 | 3.95 |
| Resolution C4 (masked, Å) | 3.42 | 3.35 | 3.46 |
| Resolution C1 (unmasked, Å) | 4.53 | 4.16 | 4.45 |
| Resolution C1 (masked, Å) | 3.90 | 3.66 | 3.84 |
|  |  |  |  |
| **Refinement** |  |  |  |
| Ramachandran |  |  |  |
| Favored (%) | 93.0 | 92.8 | 92.6 |
| Allowed (%) | 7.0 | 7.2 | 7.4 |
| Outlier (%) | 0.0 | 0.0 | 0.0 |
| Rotamer outlier (%) | 0.6 | 1.5 | 0.6 |
| Molprobity score | 1.71 | 1.93 | 1.77 |
| EMRinger score | 2.91 | 2.94 | 2.63 |
|  |  |  |  |
