# Supplementary material for: Ligand discrimination and gating in cyclic nucleotide-gated ion channels from apo and partial agonist-bound cryo-EM structures
Source: eLife. 2018 Jul 20;7:e39775. doi: 10.7554/eLife.39775 (PMC6093708; doi:10.7554/eLife.39775)
Supplement: Transparent reporting form [file elife-39775-transrepform.docx]

***eLife’s* transparent reporting form**

We encourage authors to provide detailed information *within their submission* to facilitate the interpretation and replication of experiments. Authors can upload supporting documentation to indicate the use of appropriate reporting guidelines for health-related research (see [EQUATOR Network](http://www.equator-network.org/%20)), life science research (see the [BioSharing Information Resource](https://biosharing.org/)), or the [ARRIVE guidelines](http://www.plosbiology.org/article/info:doi/10.1371/journal.pbio.1000412) for reporting work involving animal research. Where applicable, authors should refer to any relevant reporting standards documents in this form.

If you have any questions, please consult our Journal Policies and/or contact us: [editorial@elifesciences.org](mailto:editorial@elifesciences.org).

**Sample-size estimation**

- You should state whether an appropriate sample size was computed when the study was being designed
- You should state the statistical method of sample size computation and any required assumptions
- If no explicit power analysis was used, you should describe how you decided what sample (replicate) size (number) to use

Please outline where this information can be found within the submission (e.g., sections or figure legends), or explain why this information doesn’t apply to your submission:

Table S1 lists number of particles used in the reconstruction of each of the cryoEM maps. The number of separate bilayers used for single-channel recordings is disclosed in the methods section.

**Replicates**

- You should report how often each experiment was performed
- You should include a definition of biological versus technical replication
- The data obtained should be provided and sufficient information should be provided to indicate the number of independent biological and/or technical replicates
- If you encountered any outliers, you should describe how these were handled
- Criteria for exclusion/inclusion of data should be clearly stated
- High-throughput sequence data should be uploaded before submission, with a private link for reviewers provided (these are available from both GEO and ArrayExpress)

Please outline where this information can be found within the submission (e.g., sections or figure legends), or explain why this information doesn’t apply to your submission:

In cryo electron microscopy, we use many particles and merge multiple data sets.

As shown in table S1, the number of particles after 2D classification is much larger than the one used for the 3D reconstruction. As everything that physically exists on the grid is imaged with an electron microscope, we need to remove those particles that are not representing the protein under study (dust, gold, fragmented proteins, empty nanodiscs), and this is done by size and shape exclusion.

In electrophysiology, many recordings were obtained from different channels. All channels behave similarly, as stated in the methods section.

**Statistical reporting**

- Statistical analysis methods should be described and justified
- Raw data should be presented in figures whenever informative to do so (typically when N per group is less than 10)
- For each experiment, you should identify the statistical tests used, exact values of N, definitions of center, methods of multiple test correction, and dispersion and precision measures (e.g., mean, median, SD, SEM, confidence intervals; and, for the major substantive results, a measure of effect size (e.g., Pearson's r, Cohen's d)
- Report exact p-values wherever possible alongside the summary statistics and 95% confidence intervals. These should be reported for all key questions and not only when the p-value is less than 0.05.

Please outline where this information can be found within the submission (e.g., sections or figure legends), or explain why this information doesn’t apply to your submission:

Examples of the raw data for the single-channel recordings are given in Figures 1E and 2E.

Examples of the raw data for the cryoEM are given in Fig. S2-S4.

Resolution was estimated by Fourier shell correlation (FSC) between two half maps independently processed from two randomly assigned particle stacks. Correlation threshold was FSC=0.143 which is standard in the field.

Model overfitting was assessed by randomly displacing all atoms by 0.5A and real space refinement against half map 1. FSCs were calculated between the refined model and half map 1, half map 2 and the sum of both half maps.

Information can be found in Fig. S2-S4 and in the method section

(For large datasets, or papers with a very large number of statistical tests, you may upload a single table file with tests, Ns, etc., with reference to sections in the manuscript.)

**Group allocation**

- Indicate how samples were allocated into experimental groups (in the case of clinical studies, please specify allocation to treatment method); if randomization was used, please also state if restricted randomization was applied
- Indicate if masking was used during group allocation, data collection and/or data analysis

Please outline where this information can be found within the submission (e.g., sections or figure legends), or explain why this information doesn’t apply to your submission:

In 3D refinement, particles were randomly distributed into two groups and processed independently. Half maps were used for resolution estimation and generation of the final maps.

Masking of the nanodisc/protein was used in focused classification, focused refinement and postprocessing (RELION).

Information is mentioned in the method section.

**Additional data files (“source data”)**

- We encourage you to upload relevant additional data files, such as numerical data that are represented as a graph in a figure, or as a summary table
- Where provided, these should be in the most useful format, and they can be uploaded as “Source data” files linked to a main figure or table
- Include model definition files including the full list of parameters used
- Include code used for data analysis (e.g., R, MatLab)
- Avoid stating that data files are “available upon request”

Please indicate the figures or tables for which source data files have been provided:
